# Characterization of molecular diversity and genome-wide mapping of loci associated with resistance to stripe rust and stem rust in Ethiopian bread wheat accessions

**DOI:** 10.1186/s12870-017-1082-7

**Published:** 2017-08-04

**Authors:** Kebede T. Muleta, Matthew N. Rouse, Sheri Rynearson, Xianming Chen, Bedada G. Buta, Michael O. Pumphrey

**Affiliations:** 1 Department of Crop and Soil Sciences, Washington State University, Pullman, WA 99164-6420 USA; 2 USDA-ARS Cereal Disease Laboratory, Department of Plant Pathology, University of Minnesota, St. Paul, MN 55108 USA; 3 USDA-ARS, Wheat Health, Genetics, and Quality Research Unit, and Department of Plant Pathology, Washington State University, Pullman, WA 99164-6430, Pullman, WA 99164-6430 USA; 4 Ethiopian Institute of Agricultural Research, Kulumsa Agricultural Research Center, P. O. Box 489, Assela, Ethiopia

**Keywords:** Bread wheat, Stripe rust, Stem rust, Genetic resistance, Genetic diversity, Association mapping

## Abstract

**Background:**

The narrow genetic basis of resistance in modern wheat cultivars and the strong selection response of pathogen populations have been responsible for periodic and devastating epidemics of the wheat rust diseases. Characterizing new sources of resistance and incorporating multiple genes into elite cultivars is the most widely accepted current mechanism to achieve durable varietal performance against changes in pathogen virulence. Here, we report a high-density molecular characterization and genome-wide association study (GWAS) of stripe rust and stem rust resistance in 190 Ethiopian bread wheat lines based on phenotypic data from multi-environment field trials and seedling resistance screening experiments. A total of 24,281 single nucleotide polymorphism (SNP) markers filtered from the wheat 90 K iSelect genotyping assay was used to survey Ethiopian germplasm for population structure, genetic diversity and marker-trait associations.

**Results:**

Upon screening for field resistance to stripe rust in the Pacific Northwest of the United States and Ethiopia over multiple growing seasons, and against multiple races of stripe rust and stem rust at seedling stage, eight accessions displayed resistance to all tested races of stem rust and field resistance to stripe rust in all environments. Our GWAS results show 15 loci were significantly associated with seedling and adult plant resistance to stripe rust at false discovery rate (FDR)-adjusted probability (*P*) <0.10. GWAS also detected 9 additional genomic regions significantly associated (FDR-adjusted *P* < 0.10) with seedling resistance to stem rust in the Ethiopian wheat accessions. Many of the identified resistance loci were mapped close to previously identified rust resistance genes; however, three loci on the short arms of chromosomes 5A and 7B for stripe rust resistance and two on chromosomes 3B and 7B for stem rust resistance may be novel.

**Conclusion:**

Our results demonstrate that considerable genetic variation resides within the landrace accessions that can be utilized to broaden the genetic base of rust resistance in wheat breeding germplasm. The molecular markers identified in this study should be useful in efficiently targeting the associated resistance loci in marker-assisted breeding for rust resistance in Ethiopia and other countries.

**Electronic supplementary material:**

The online version of this article (doi:10.1186/s12870-017-1082-7) contains supplementary material, which is available to authorized users.

## Background

Stripe rust [*Puccinia striiformis* Westend. f. sp. *tritici* Erikss. (*Pst*)] and stem rust [*Puccinia graminis* Pers.:Pers. f. sp. *tritici* Erikss. & E. Henn. (*Pgt*)] are two of the most damaging diseases of wheat worldwide [1–3]. The ability of wheat rust pathogen populations to quickly evolve new pathotypes that overcome deployed resistance genes and produce multiple cycles of urediniospores in a single season, along with their capacity for long distance dispersal, create potential for destructive epidemics in susceptible varieties under favorable conditions. At various times in history, epidemics of both diseases have caused massive losses to wheat production globally. Stripe rust has been a disease of wheat mainly in areas with cooler climates [1]. More recently, however, the emergence of aggressive races of *Pst* tolerant to higher temperatures has resulted in yield loss in areas normally considered too warm for serious epidemic development. These new strains of *Pst* are currently widespread and threatening wheat production on a global scale [1, 4, 5]. In recent years, wheat growing regions in East Africa, Central and West Asia, and the Caucasus countries experienced one of the largest stripe rust epidemics in the recent history [6]. In Ethiopia alone, this epidemic affected more than 600,000 ha of wheat and led to an expenditure of more than $US 3.2 million on fungicides, while significant widespread losses were still realized [7].

During the last 40 years, introgression of different combinations of stem rust resistance genes and other epidemic mitigation strategies have reduced global stem rust epidemics [8, 9]. However, the discovery of race TTKSK (isolate Ug99) of *Pgt* in Uganda in 1998 has raised a particular concern to global wheat production and food security due to its wide virulence spectrum [10]. Currently, at least eight variants of TTKSK (the Ug99 lineage races) have been described with virulence for additional resistance genes, including *Sr24*, *Sr36*, *Sr9h* and *SrTmp* [11–14]. These races are currently spreading across eastern, southern and northern Africa as well as the Middle East [8, 14, 15]. In 2013–2014, a new stem rust race designated as TKTTF caused a severe epidemic on the variety ‘Digalu’ carrying *SrTmp* in Ethiopia [16]. The continued emergence of new virulent races of stem rust emphasizes the dynamic challenges of breeding for stem rust resistance.

To mitigate losses due to rust diseases in wheat, world wheat production has largely depended on the use of resistant wheat varieties [1, 17, 18]. The emergence of pathogen populations virulent against deployed resistance genes has caused not only tremendous yield and quality losses, but has also led to frequent replacement of the otherwise agronomically superior cultivars, as well as interference with progress in improving other important traits [17]. Achieving durable varietal performance has therefore been the primary focus of many breeding programs in wheat. Two categories of resistance genes have been widely recognized in wheat breeding for rust resistance; all-stage resistance (also called seedling resistance) and adult-plant resistance (APR) [1]. Developing cultivars carrying effective seedling resistance in combination with APR genes is more desirable to minimize the damage caused by new mutants of the pathogen [3, 17]. The deployment of cultivars with durable rust resistance in wheat is particularly desirable in regions such as Ethiopia and the neighboring countries in East Africa that are characterized by the presence of year-round inoculum due to nearly constant wheat cropping seasons and a suitable environment. These conditions provide the pathogen not only with a continuous substrate both in area and in time due to a green bridge between the seasons, but may also result in rapid selection for virulent races of the pathogen.

Limited variation in elite germplasm may constrain deployment of diverse resistance genes in released cultivars and the capacity for countering new virulence in pathogen populations [19, 20]. Landraces and other germplasm collections maintained in germplasm banks may provide access to diverse alleles for disease resistance and reverse the trend of genetic diversity erosion in established, elite cultivars [21, 22]. Such genetic resources are potentially untapped sources of useful genetic diversity owing to their limited use in modern plant breeding programs. The usefulness of wheat landraces as a good source of resistance to diseases in wheat have been demonstrated [23–27]. The objectives of this study were: 1) to evaluate Ethiopian bread wheat landraces and cultivars for their field and seedling resistance to stripe rust and stem rust across a range of environments and multiple races of the pathogens, 2) to assess the genetic diversity of Ethiopian bread wheat landraces and cultivars based on high-density genotyping, and 3) to conduct a genome-wide search for molecular markers associated with loci underpinning seedling and field resistance to stripe rust and stem rust diseases of wheat.

## Methods

### Plant materials

One hundred and ninety bread wheat accessions were used in this study. The panel was comprised of 124 landraces and 66 commercial cultivars and advanced breeding lines from the Ethiopian Institute of Agricultural Research (EIAR). Most of the commercial cultivars and advanced breeding lines were originally developed by the International Maize and Wheat Improvement Center (CIMMYT), some by the International Center for Agricultural Research in the Dry Areas (ICARDA), but evaluated and released as cultivars by the Ethiopian wheat improvement program. One hundred and ten accessions were landraces of hexaploid wheat originally from the Ethiopian Institute of Biodiversity, characterized and maintained by EIAR at Debre-Zeit Agricultural Research Center (DZARC), from where they were obtained. Fourteen landraces were obtained from the United States Department of Agriculture, Agricultural Research Service (USDA-ARS) National Small Grains Collection (NSGC) in Aberdeen, ID, USA. Two susceptible cultivars, Avocet S and Morocco, used in this study as checks were obtained from the USDA-ARS, Wheat Health, Genetics, and Quality Research Unit, Pullman, WA; and DZARC, respectively.

### Stripe rust response evaluation under field conditions

The panel was tested for response to stripe rust infection under field conditions in four nurseries in the Pacific Northwest (PNW) of the US and in Ethiopia for three consecutive growing seasons (2012–2014). The locations in the PNW were Mount Vernon (48° 25′ 12″N; 122° 19′ 34″W), a high rainfall area located west of the Cascade Mountain range; and Pullman (46° 43′ 59″ N; 117° 10′ 00″W), a semi-arid wheat belt area located east of the Cascade Mountain range. Locations in Ethiopia included Meraro (07^○^24′27″ N; 39^○^14′56″ E) and Arsi Robe (07°53′02″ N; 39°37′40″ E), sub-stations of Kulumsa Agricultural Research Center of the Ethiopian Institute of Agricultural Research, representing relatively high annual rainfall and cool temperatures. Each nursery location is subject to high disease pressure on an annual basis, but has variable *Pst* races and environmental conditions. The accessions were planted as single rows of non-replicated trials with repeating checks. The susceptible cultivars Avocet S (at Mount Vernon and Pullman) and Morocco (at Meraro and Arsi Robe) were planted every 20 rows and on each side of the plot to ensure uniform disease pressure across the experimental plots. Accessions were evaluated for response *Pst* under natural disease epidemics. Host response to infection [i.e. infection types (IT)] to stripe rust was estimated using a 0–9 scale as described by Line and Qayoum [28]. Stripe rust disease severity (DS) was recorded as percent leaf area showing disease symptoms. No permits were required to carry out field experiments. In addition, the field studies did not involve endangered or protected species.

### Seedling resistance screening to Pst

Seedlings of the 190 accessions were evaluated for response to five isolates representing five races of *Pst* under greenhouse conditions at Washington State University, Pullman, WA. Three of the races (PSTv-14, PSTv-37 and PSTv-40) were of US origin [29], one race from Ethiopia, but also detected in the USA (PSTv-41) and one race predominant only in Ethiopia (PSTv-106) were used in the study [30]. The virulence/avirulence formulae of the isolates are presented in Additional file 1. All *Pst* races used in this study were obtained from the USDA-ARS, Wheat Health, Genetics, and Quality Research Unit, Pullman, WA.

For the seedling test, three to five seeds of each accession were planted into pots filled with the Sunshine® mix growing medium (Sun Gro Horticulture, Agawam, MA, and USA). The susceptible check, ‘Avocet S’ and the stripe rust single-gene differential lines were also included in each experiment. Ten-day old seedlings were inoculated with urediniospores of each race. The urediniospores were mixed with talcum to ensure uniform coverage of the spores over the leaves of the seedlings. Inoculated seedlings were kept in a dark dew chamber for 24 h at 10 °C, and then transferred into a growth chamber with temperature programmed to change gradually between 4 °C at 2:00 am during the 8 h dark period and 20 °C at 2:00 pm during the 16-h light period [29]. Stripe rust infection types (IT) based on the 0–9 scale were recorded at 18–20 days after inoculation as described by Line and Qayoum [28].

### Seedling resistance screening to Pgt

The Ethiopian bread wheat cultivars and landraces were evaluated for reaction to four *Pgt* races at the USDA-ARS Cereal Disease Laboratory (CDL), St. Paul, MN. The four stem rust races were TTKSK (isolate 04KEN156/04), TRTTF (06YEM34–1), TTTTF (01MN84A-1-2) and TKTTF (13ETH18–1) that were selected based on their differential virulence patterns and potential impact on wheat production. TRTTF is broadly virulent to stem rust resistance genes including *Sr1RS* ^*Amigo*^ and was detected in Yemen and Ethiopia [31]. Race TTKSK (Ug99) has a wide virulence spectrum and is rapidly evolving in East Africa. Race TTTTF is the most virulent race from the United States, producing high infection types (ITs) on the majority of stem rust differential lines [32]. TKTTF is a new race that was responsible for the localized severe stem rust epidemic in Ethiopia in 2013–2014 due to its virulence to the stem rust resistance gene *SrTmp* in cultivar ‘Digalu’ [16]. Seedling resistance evaluations were performed as described previously by Rouse et al., 2011 [33]. Infection types (ITs) were recorded on a 0 to 4 scale according to Stakman et al., 1962 [34]. Stakman ITs were converted to a linear scale using a conversion algorithm [35]. All the *Pgt* races used in this study were obtained from the USDA-ARS Cereal Disease Laboratory (CDL), St. Paul, MN.

### Genotyping

The 190 Ethiopian bread wheat accessions were genotyped using the 90,000 SNP Illumina Infinium assay at USDA-ARS Fargo, ND, USA. Illumina genotypic data were analyzed using Illumina GenomeStudio v2011.1 software to optimize the SNP call rates. After removing SNPs with low-quality clustering and those with minor allele frequency (MAF) less than 5%, 24,281 high quality SNP markers with genetic map information were used for GWAS analyses. The genetic positions of the SNP markers were based on the wheat 90 K SNP consensus map [36]. The dataset was also filtered using a 10% cutoff for missing data in the accessions. Accordingly, a total of 179 accessions were retained in the GWAS and population structure analyses.

For internal controls, the accessions were screened with molecular markers for known stripe rust and stem rust resistance genes, including *Lr34/Yr18/Pm38* (KASP marker wMAS000003) [37], *Lr37/Yr17/Sr38* (CAPS marker Ventriup-LN2) [37], *Lr67/Yr46* (Kasp856) [38], *Sr31/Yr9/Lr26* (KASP 1RS:1BL_6110) [39], *Sr24* (barc71) [40], *Sr36* (KASP wMAS000015), [37], *Sr2/Yr30* (KASP wMAS000005) [37]. Markers with a MAF greater than 5% were included in the GWAS and linkage disequilibrium analyses.

### Analyses of molecular diversity and population structure

Genetic diversity was analyzed based on Nei’s gene diversity, which is an estimate of the probability that two randomly chosen alleles from the population are different, and polymorphism information content (PIC), which reflects the probability of polymorphism between two random samples in the germplasm, using POWERMARKER v3.25 [41, 42]. The two indices of genetic diversity were used to compare the extent of molecular diversity among the landrace collection and the contemporary Ethiopian bread wheat cultivars. A *t*-test was performed using the software package JMP Genomics v6.0 (SAS Institute, Cary, NC) to compare the extent of molecular diversity between landraces and elite Ethiopian cultivars.

Cluster analysis based on the neighbor joining (NJ) tree algorithm according to shared-allele distance was also used to determine the genetic structure of the accessions using the phylogenetic tree analysis package in POWERMARKER v3.25. The assessment of the branching pattern in the NJ tree was based on bootstrapping over loci with 1000 replications. FigTree program v1.4 (http://tree.bio.ed.ac.uk/software/figtree/) was used to display the consensus bootstrap value generated by PHYLIP v3.66 [43].

Population structure was analyzed using a set of 355 random SNP markers distributed across the 21 wheat chromosomes (12–23 markers per chromosome) spaced at >10 cM apart. The admixture model with correlated allele frequency implemented in STRUCTURE software version 2.2.3 was applied to detect population structure in the germplasm panel [44]. The parameters were set to a burn-in of 20,000 iterations and 50,000 Monte Carlo Markov Chain (MCMC) replicates to determine K values in the range of 1 to 10. For each K, five independent runs were carried out. The Evanno method [45] was used to determine the likely number of subpopulations. Principal components (PC) were inferred using the software package JMP Genomics v6.0 (SAS Institute, Cary, NC) to further analyze population sub-structuring and compare the results with results from analysis with STRUCTURE software.

### Linkage disequilibrium analysis

Pairwise measures of linkage disequilibrium (LD) between SNP markers were estimated using the software package JMP Genomics v6.0 (SAS Institute, Cary, NC). LD was estimated as squared allele frequency correlation (*r* ^*2*^) between pairs of intra-chromosomal SNPs with known chromosomal position. To determine the average pattern of genome-wide LD decay over genetic distance, a scatterplot of *r* ^*2*^ values against the corresponding genetic distance between markers was constructed. The second-degree locally weighted polynomial regression (LOESS)-based curve was fitted to estimate the extent of LD decay [46]. The critical *r* ^*2*^ value that indicates the demarcation beyond which LD is due to true physical linkage was determined by taking the 95th percentile of the square root of transformed *r* ^*2*^ data of unlinked markers [47]. The genetic distance at which the LD decay curve intersects with the critical *r* ^*2*^ value was used as a threshold to determine the confidence interval of significant QTL.

### Analyses of variance and heritability

Variance components were estimated for stripe rust IT and DS from the field experiments with a mixed linear model using the Restricted Maximum Likelihood (REML) method [48]. Genotype, location, genotype x location interaction and replication (season) were considered to have random effects whereas the overall mean was considered to have fixed effect. Variance components were estimated for each location and across all locations according to the following model:

$$ {y}_{ijk}=\mu +{g}_i+{\mathrm{k}}_j+{e}_j+{ge}_{ij}+{e}_{ij} $$

Where *y*_*ij*_ is the observation for genotype *i* at environment *j* in season k, *μ* is the overall mean, *g*_*i*_ the effect of the accession *i*, *e*_*j*_ the effect of environment *j*, k_*j*_ the effect of season *k*, *g*_*eij*_ the interaction between accession *i* within environment *j*, and *e*_*ij*_ the residual.

Heritability (*H* ^*2*^) estimates were calculated for each location and across all locations as:

$$ {H}^2={\sigma^2}_G/\left({\sigma^2}_G+\left({\sigma^2}_E/y\right)+\left({\sigma^2}_{GXE}/y\right)+{\sigma^2}_{error}/y\right) $$

Where *σ* ^*2*^_*G*_ is the genotypic variance, *σ* ^*2*^_*E*_ is the environment variance, *σ* ^*2*^_*GXE*_ is the genotype by environment interaction variance, and *σ* ^*2*^_*error*_ is the residual error variance and y is the number years within each location or location by year for the estimates of heritability across all environments. Genotype adjusted means were computed based on best linear unbiased predictions (BLUPs). Pearson correlation coefficient between locations and seasons were calculated to determine the consistency of IT and DS across the environments.

### Marker-trait association (MTA)

To identify loci associated with response to *Pst* and *Pgt*, genome-wide association analyses were conducted using a total of 24,281 high quality SNP markers and phenotypic traits values (IT and DS) from the field and greenhouse experiments. Marker-trait associations (MTAs) were identified using the compressed mixed linear model (CMLM) [49, 50] implemented in GAPIT (Genomic Association and Prediction Integrated Tool) R package [51]. The first three principal components (PC3) and a compressed relationship matrix (K) were included as fixed and random effect, respectively. Three additional association test models were compared to the PC3 + K CMLM model for correcting population structure and kinship based on the deviances of observed probability from expected distribution in Q-Q plot were used to compare the models. The following three additional models were tested: a fixed general linear model with no correction for population structure (GLM), general linear model with the first three principal components included as a fixed covariate (PC3 GLM) and mixed linear model with compressed relationship matrix included as a random covariate (K CMLM). Association analyses were conducted for IT and DS values from each location separately and estimates of BLUPs for each location and across all environments.

## Results

### Phenotypic variability and estimates of heritability

**Fig. 1:**
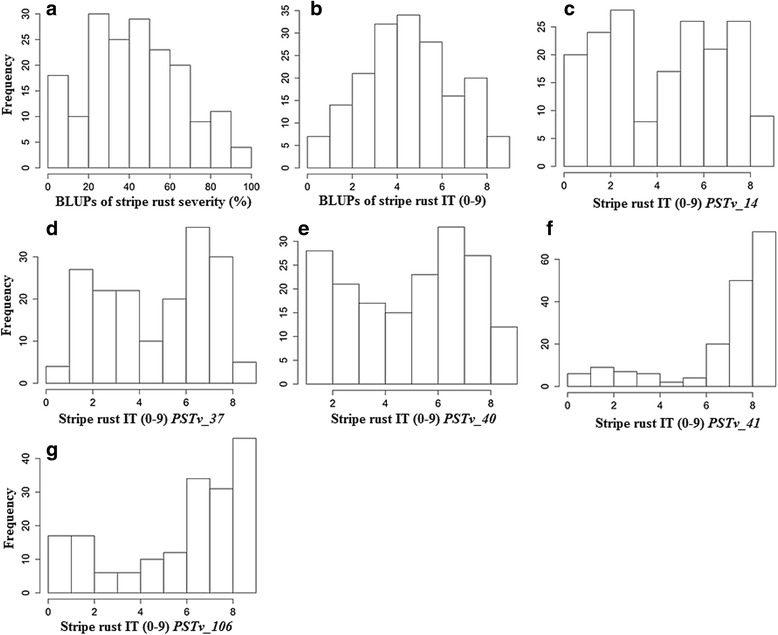
Frequency distributions of stripe rust responses produced by the Ethiopian wheat accessions. **a** and **b** stripe rust infection types (IT) and disease severity (DS) under field conditions, **c**-**g** ITs produced by seedlings when tested against the five races of *Pst* under greenhouse conditions

Analysis of variance revealed a highly significant (*P* < 0.0001) difference among the genotypes both for individual locations and data combined across locations (Table 1). Genotype by environment interactions were significant (*P* < 0.01) for disease severity at Pullman, Mount Vernon and the combined analysis across all locations. The variance components for locations were not significant across all analyses. Based on the IT data, 21 accessions (11%) were highly resistant to all races of *Pst* as seedlings and across all locations and seasons at adult plant stages. Eleven of the 21 resistant accessions were landraces, while the remaining 10 accessions were cultivars. Twenty-three accessions (13%) showed highly susceptible reactions to all races as seedlings and across all locations and seasons at adult plant stage (Fig. 1). Heritability (*H* ^*2*^) values for stripe rust IT and DS ranged from 73% to 93% (Table 1). Correlation coefficients between locations and seasons for stripe rust IT and DS are summarized in Additional file 2. The Pearson correlation coefficients for stripe rust IT and DS between the multiple locations over multiple growing seasons averaged 0.64 and 0.72, respectively. Average correlations between seasons within locations were 0.79, 0.76 and 0.64 for IT, and 0.80, 0.86 0.68 for DS at Pullman, Mount Vernon and Ethiopia, respectively. Correlation between Pullman and Mount Vernon (over multiple seasons) (averaged 0.72 and 0.78 for IT and DS, respectively), were significantly higher than the correlation between Ethiopia and the two locations in PNW; the respective average correlation coefficients for IT and DS were 0.60 and 0.58 between Pullman and Ethiopia, and 0.54 and 0.55 between Mount Vernon and Ethiopia.

**Table 1:** Mean response to *Puccinia striiformis* f. sp. *tritici* infection, estimates of variance components and heritability

Parameter	Pullman		Mount Vernon		Ethiopia	Across locations	
	IT (0–9)	Severity (%)	IT (0–9)	Severity (%)	IT (0–9)	IT (0–9)	Severity (%)
Minimum	0.0	0.0	0.0	0.0	0.0	0.0	0.0
Mean	4.2	41.0	4.2	43.0	4.8	4.4	42.0
Maximum	9.0	100.0	9.0	100.0	9.0	0.0	100.0
σ^2^_G_	2.7^***^	360.0^****^	4.5^***^	612.0^****^	4.1^***^	2.9^***^	451.2^****^
σ^2^_E_	0.9^ns^	141.0^ns^	0.1^ns^	4.4^ns^	0.1^ns^	0.3^ns^	48.1
σ^2^_GXE_	0.1^ns^	117.0^***^	0.0	90.9^**^	0.3^*^	0.2^*^	136.9^***^
σ^2^_error_	1.0	1.0	0.7^***^	1.0	1.0	1.8	1.0
Heritability	0.73	0.74	0.92	0.93	0.85	0.89	0.91

${\sigma}_G^2$ = estimate of genotypic variance

${\sigma}_E^2$ = estimate of environmental variance

${\sigma}_{GE}^2$ = estimate of genotype x environment variance

${\sigma}_e^2$ = estimate of residual variance; *H* ^2^ = heritability

*IT* infection type; *DS* disease severity; *ns* not significant

*P < 0.05

**P < 0.01

***P < 0.001

****P < 0.0001

**Fig. 2:**
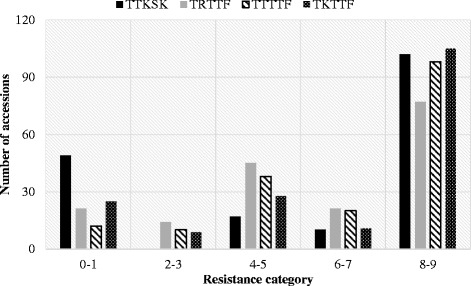
Frequency distributions of stem rust infection types produced by seedlings of the Ethiopian wheat accessions

The frequency distribution of the linearized ITs produced by the four *Pgt* races on the Ethiopian cultivars and landraces is shown in Fig. 2. Among all accessions, 75 (41%), 84 (46%), 61 (34%) and 69 (38%) showed seedling resistance to races TTKSK, TRTTF, TTTTF and TKTTF, respectively. Fifteen (8%) of the accessions were resistant (IT = 0 to 23) to all four races, of which the majority were landraces. Eight accessions (5%) shared resistance to the four races of *Pgt* and field resistance to *Pst* at all environments. Five of the 8 accessions resistant to the four races of *Pgt* and with field resistance to *Pst* at all environments were cultivars, while the remaining 3 were landraces. Additional file 3 summarizes the responses of the accessions to *Pst* and *Pgt* infection in the seedling and field resistance screenings.

### Molecular diversity and population structure

**Fig. 3:**
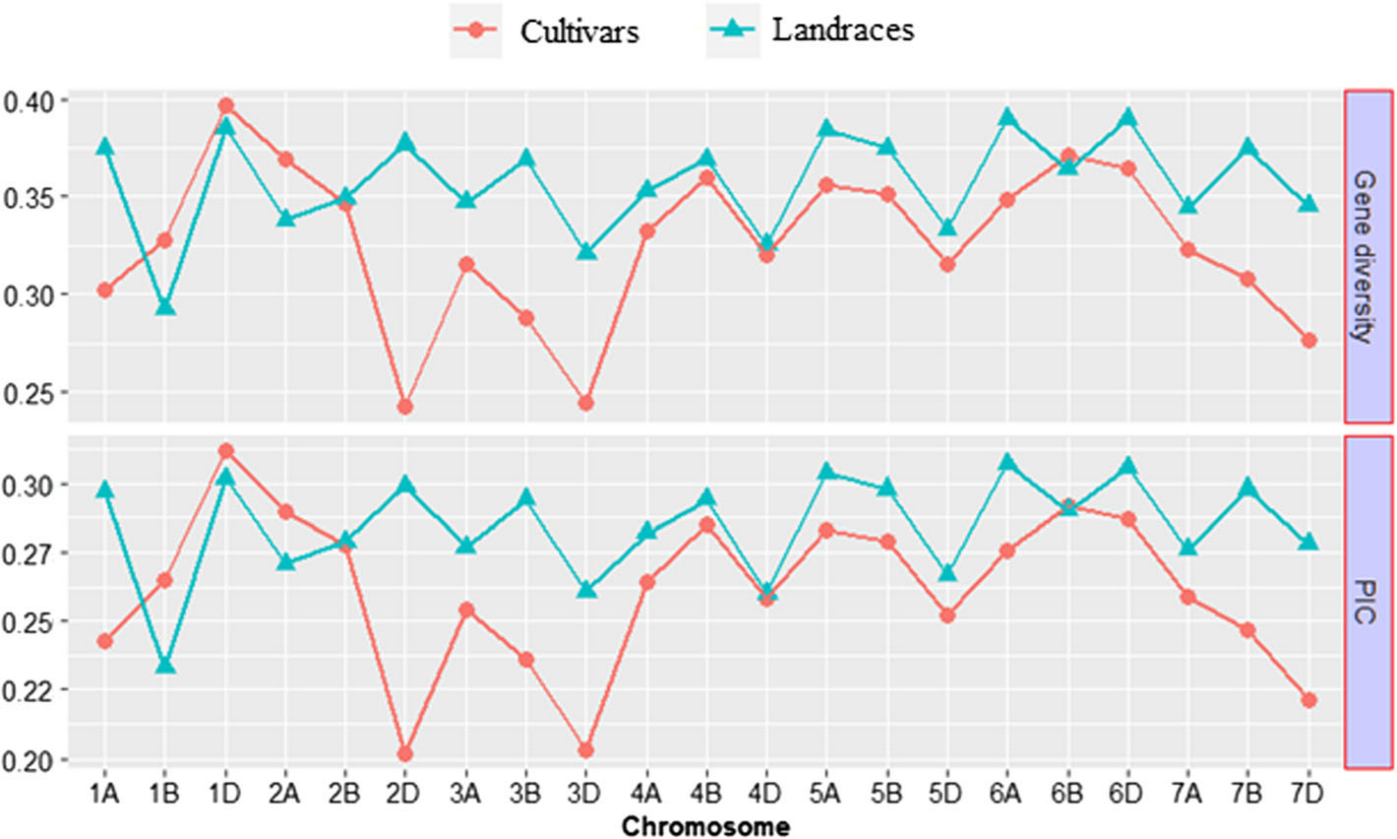
Genome specific comparisons of molecular diversity between elite cultivars and gene bank conserved landrace accessions. Nei’s gene diversity and polymorphism information content (PIC) values were used to compare the extent of genetic variation in modern Ethiopian cultivars and landrace accessions

Genome-specific analyses of Nei’s gene diversity and PIC values were used to compare the extent of genetic variation in modern Ethiopian cultivars and accessions maintained in the germplasm collection. The analyses revealed a highly significant difference (*P* < 0.001) between cultivars and landraces in terms of both Nei’s gene diversity index and PIC values. Both diversity indices were significantly higher in the landraces in 13 of the 21 wheat chromosomes (including 1A, 2D, 3A, 3B, 3D, 4A, 5A, 5B, 6A, 6D, 7A, 7B and 7D). The cultivars showed higher values of gene diversity and PIC values over the landraces only in chromosome 1B and 2A. Genome-wide average Nei’s gene diversity index values were 0.36 and 0.32 for the landraces and cultivars, respectively, while the PIC values were 0.29 and 0.25 for the landraces and cultivars, respectively (Fig. 3).

**Fig. 4:**
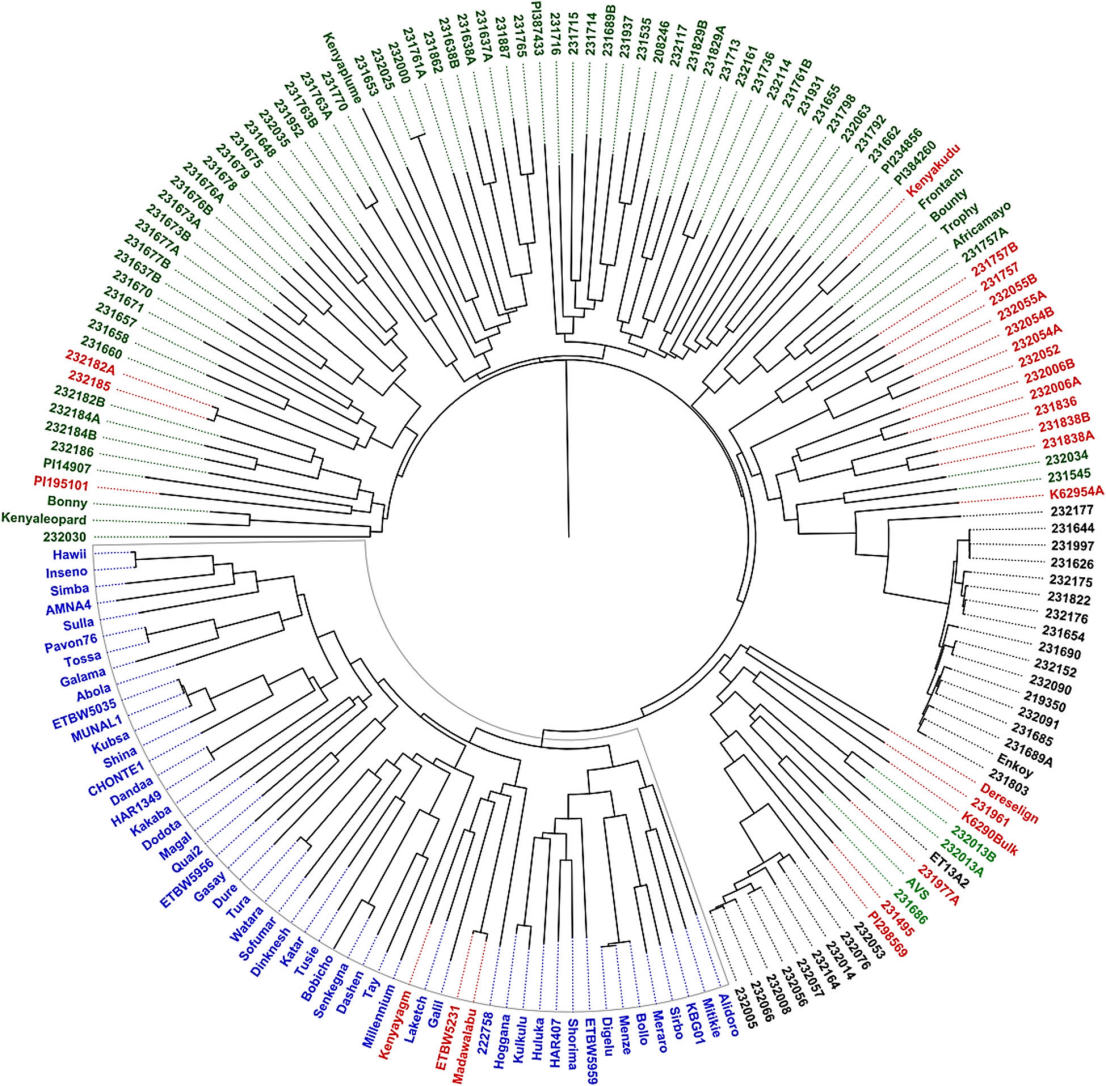
Dendrogram of the Ethiopian wheat accessions estimated by shared-allele genetic distance using high-density SNP markers. Cluster analysis was based on the neighbor-joining algorithm. Accessions have been assigned colors based on STRUCTURE analysis at K = 3. *Black* = sub-population 1 (SP_I), *Green* = sub-population 2 (SP_II (i)) and *Blue* = subpopulation 3 (SP_II (ii)). Structure analysis indicated the likely number of sub-population to be two (SP_I and SP_II). Separate analysis of population structure within SP_I showed no sign of further sub-division, while SP_II subdivided into SP_II (i) and SP_II (ii). The blue colored accessions (SP_II (ii) in the structure analysis) are Ethiopian cultivar*s, while the rest are landraces*

A neighbor-joining (NJ) phylogeny analysis based on shared allele distance showed that the Ethiopian improved varieties exhibited a high degree of genetic relatedness and were clearly distinct from the majority of landraces (Fig. 4). Based on pairwise kinship analysis using the Fast Ward clustering algorithms, about 31% of the released commercial cultivars showed coefficient of kinship greater than 0.9 (1.0 is exact resemblance) with each other.

**Fig. 5:**
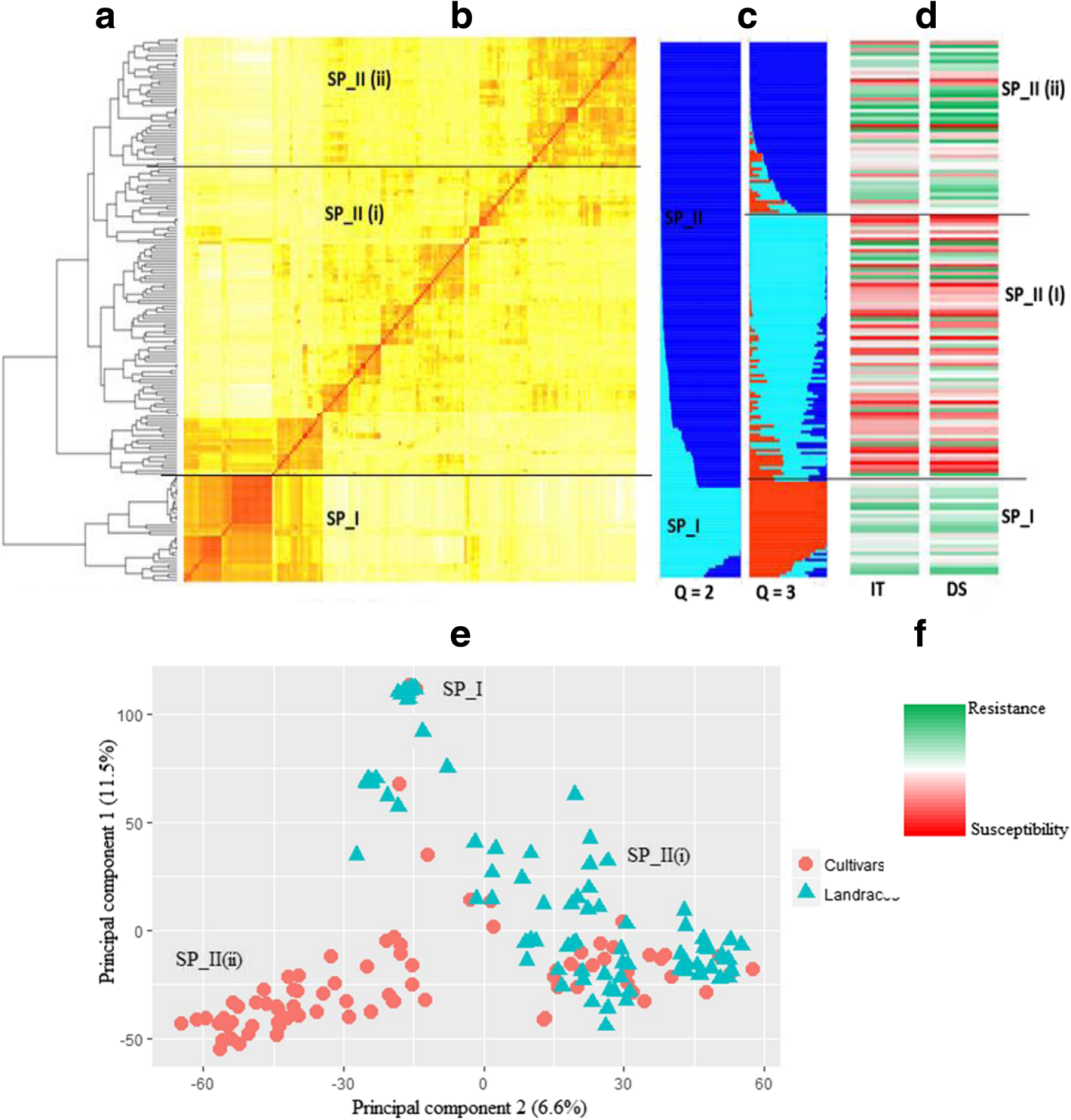
Genetic relatedness, population structure and the relationship between population sub-clustering and stripe rust resistance. **a** The distance based hierarchical clustering of the accessions based on the Fast Ward grouping algorithm. **b** Heat map of identity-by-decent (IBD) kinship matrix. **c** Clustering diagrams of population structure based on the model based quantitative assessment of subpopulation membership. **d** Heat map of reactions of the accessions to stripe rust IT and DS based on BLUP values across all environments. **e** Principal component analysis (PCA). **f** Color key for the heat map of the phenotypes. SP_I and SP_II are sub-populations defined according to the optimum number of clusters determined by STRUCTURE analysis. SP-II was further sub-grouped into two (SP-II (i) and SP-II (ii)) based on kinship analysis that agrees with the STRUCTURE analysis when K = 3 was considered. Accessions in SP-II (ii) are all recent and historical Ethiopian bread wheat cultivars

Bayesian analysis of population structure yielded a strong signal for the existence of two distinct genetic clusters in the germplasm panel. With K = 2, more than 91% of the accessions were assigned to a specific cluster with membership probability higher than 75%, with the remaining accessions (9%) being classified as admixed. Kinship and principal component analysis (PCA) tended to group the accessions into three subgroups, in which all of the improved varieties were categorized into a distinct third cluster (Fig. 5 **)**. The structure analysis yielded similar clustering patterns as PCA when the second subpopulation was further analyzed separately. PCA revealed that the first and second principal component PCs accounted for 11.5% and 6.6% of the variance, respectively.

### Linkage disequilibrium estimation

**Fig. 6:**
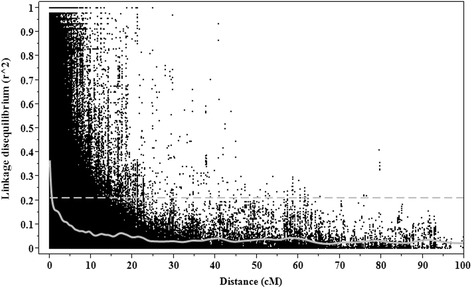
Genome-wide linkage disequilibrium decay plot for the Ethiopian wheat accessions based on high-density SNP markers. LD, measured as *r* ^*2*^ between pairs of polymorphic marker loci is plotted against the genetic distance (cM) between the markers. LOESS smoothening curve (*solid line*) and mean LD (*broken line*) were fitted to the LD decay

The extent of LD and average rate of LD decay were estimated by squared correlation coefficient (*r* ^*2*^) for all pairs of SNPs along each chromosome. LD due to physical linkage, estimated as the 95th percentile of the distribution of LD *r* ^*2*^ between unlinked SNPs, was 0.18. On a genome-wide level, 33.3% of all pairwise SNP loci showed LD greater than the critical value (*r* ^*2*^ > 0.18). In the A genome, the percentage of marker pairs that were in LD greater than 0.18 for chromosome 1A was the highest (6.8%), while in the B genome chromosome 1B contained the highest percentage of marker pairs (11.0%) that showed LD greater than the critical *r* ^*2*^ value. For the D genome, the percentage of SNP pairs that showed LD greater than 0.18 for chromosome 2D was the highest (4.2%). LD decayed to the critical *r* ^*2*^ value (0.18) at about 2.5 cM, which was used to determine the confidence interval for declaring distinct MTAs (Fig. 6).

### Association analysis for field-based and seedling resistance to Pst

Genome-wide association analyses were performed for stripe rust IT and DS within each of the eight environments. Based on the assessment of the performance of the GWAS models for controlling population structure and kinship, the K + PC3 CMLM model (containing the first three principal components and a compressed kinship) gave a minimum deviance of observed *P* values from expected distribution in Q-Q plot (Additional file 4) and was used for all GWAS analyses. The analyses revealed 67 genomic regions significantly associated with adult plant IT or DS at nominal probability (*P*) < 0.005 in at least four test environments. A confidence interval for linkage blocks of the 67 regions was determined based on average LD decay rate (±2.5 cM) and adjacent markers were assigned to the linkage blocks based on the significance and consistency of associations across environments. Markers with the most significant associations to each trait were used as the MTA-tagging marker. Altogether, the 67 genomic regions explained 54.3% and 52.1% of the total variation in IT and DS, respectively (excluding variation explained by the population structure). Additional file 5 summarizes detailed information of the 67 putative resistance loci.

Eleven of the 67 genomic regions were significant at False Discovery Rate (FDR) [52] adjusted *P* < 0.1 (Table 2). The 11 high-probability QTL were located on chromosomes 1B, 2A, 4A, 5A, 6A and 7B. The phenotypic variation (*R* ^*2*^) explained by these genomic regions were in the range of 3–11%. When combined, these loci explained 27.5 and 26.9% of the total variation in *Pst* IT and DS responses, respectively. Results of the GWAS tests are discussed in detail focusing on the chromosome regions with significance at FDR-adjusted *P* < 0.1.

**Table 2:** Chromosomal location, probability of association (*P*) and *R* ^*2*^ values of SNPs markers representing genomic regions significantly associated (FDR adjusted *P* < 0.1) with stripe rust infection type (IT) and disease severity (DS) from field experiments and seedling reactions against five races of the stripe rust pathogen in the Ethiopian bread wheat accessions

QTL-tagging SNP index^a^	SNP^b^	Associated SNP index^c^	Chr.	Position (cM)^d^	RAF (%)	*P* values (−log)^e^	*R* ^*2*^ (%)	Traits/*Pst* races^f^	Previously mapped *Yr* genes/QTL^g^
*Field experiments*									
*IWB11553*	T/G	*IWB63258, IWB42176*	1B	64.9	8.4	2.6–6.3	3.1–9.6	PLM_IT_12, MTV_DS_13, MTV_IT_13, MTV_IT_14	*Yr15, Yr24, YrAlp, Yr64, YrCH52, Yr65, QYr.caas-1BL.1RS_SHA3/CBRD*
*IWB9661*	A/G	*IWB20091*	1B	74.4	7.0	2.7–4.6	3.4–6.7	*PSTv-37*, PLM_IT_12	*QYr.cim-1BS_Pastor*
*IWB11136*	T/G	*IWB22615, IWB71860*	2A	9.4	6.7	2.5–5.2	3.5–8.3	ETH_IT_12, ETH_IT_14, ETH_DS_14, PLM_IT_12, PLM_DS_12	*Yr17, Yr56, QYr.tam-2AS_TAM111, QYr.uga-2AS_26R61, QYr.ufs-2AS_Cappelle Desprez*
*IWB6584*	A/G	*IWB42693*	2A	26.0	10.6	2.4–7.3	3.1–11.2	*PSTv-41*, ETH_IT_12, ETH_IT_14, PLM_IT_12, PLM_DS_12	
*IWB57199*	T/C	*IWB63394*	2A	47.2	9.5	2.6–6.8	3.4–9.5	*PSTv-41,* ETH_IT_12, ETH_IT_14, ETH_DS_14, PLM_IT_12, PLM_DS_12	*QYrva.vt-2AS_VA00W-38, QYr.inra-2AL_CampRemy*
*IWB24187*	T/C	*-*	4A	114.5	19.0	2.3–4.3	2.8–6.1	MTV_DS_12, MTV_IT_14	*Yr51*, *QYr-4A_Sachem, QYrst.orr-4AL_Stephens*
*IWA1280*	A/G	*IWA7226, IWA8119, IWB11086, IWB58658*	5A	15.7	17.7	2.1–4.6	2.7–6.1	PLM_DS_12, PLM_IT_14, ETH_IT_14	*_*
*IWB28837*	T/C	*IWB79251*	5A	89.6	44.7	2.6–4.5	3.0–7.1	MTV_DS_12, MTV_IT_13	*_*
*IWB69846*	A/G	*_*	6A	136.9	10.6	2.4–5.5	3.1–8.3	PLM_DS_12, PLM_IT_12	*QYr-6A_Avocet, QYr-6A_Saar, QYr.ufs-6A_Kariega, YrLM168, QYr.cim-6AL_Francolin*
*IWA3506*	A/G	*IWB33903, IWB39783*	7B	56.9	64.8	2.6–5.2	3.1–8.1	PSTv-41, PLM_DS_14, MTV_IT_14, PLM_IT_12	*_*
*IWB58601*	A/G	_	7B	155.4	5.0	2.3–5.3	3.1–8.4	PLM_IT_14, MTV_DS_14	*Yr59, YrC591, Yr52, Yr67, YrZH84, QYr7BL_Strongfield, QYr.cim-7BL_Pastor*
*Seedling resistance*									
*IWB9661*	A/G	*IWB20091*	1B	74.4	7.4	5.9	13.2	*PSTv-37*, PLM_IT_12	*QYr.cim-1BS_Pastor*
*IWB6584*	A/G	*IWB42693*	2A	26.0	10.4	4.4	9.4	*PSTv-41*, ETH_IT_12, ETH_IT_14, ETH_DS_14, PLM_IT_12, PLM_DS_12	*Yr17, Yr56, QYr.tam-2AS_TAM111, QYr.uga-2AS_26R61, QYr.ufs-2AS_Cappelle Desprez*
*IWB57199*	T/C	*IWB57200*	2A	47.2	11.0	4.9	10.6	*PSTv-41*, ETH_IT_12, ETH_IT_14, ETH_DS_14, PLM_IT_12, PLM_DS_12	*QYrva.vt-2AS_VA00W-38, QYr.inra-2AL_CampRemy*
*IWB43797*	T/**C**	*IWB29589, IWB81562, IWB32481*	2B	134.5	33.5	6.0	13.7	*PSTv-41*, *PSTv-106*	*Yr43*
*IWB10441*	T/**G**	–	2D	82.8	12.5	6.6	11.9	*PSTv-106*	*QPst.jic-2D_Guardian*
*IWB14332*	T/**C**	–	5B	29.1	45.4	4.6	9.7	*PSTv-40*	*Yr47, QYr.uga-5B_AGS2000*
*IWB48375*	**T**/C	*IWB81061, IWB78446*	5B	71.9	38.4	6.2	11.7	*PSTv-14*	*YrEXP2, QYr.caas-5BL.1_Libellula, QYr-5B_Oligoculm*
*IWA3506*	A/G	*IWB33903, IWB39783*	7B	56.9	64.8	2.6–5.2	3.1–8.1	*PSTv-41*, PLM_DS_14, MTV_IT_14, PLM_IT_12	*_*

^a^SNP index from the wheat 90 K iSelect assay. Within the confidence interval of the QTL, marker with the most significant associations were used as the QTL-tagging marker

^b^Underline indicates favorable allele

^c^Other significant SNPs identified within the confidence interval of the QTL other than the QTL-tagging marker

^d^Scaled position from hexaploid wheat consensus map (Wang et al. 2014)

^e^Marker-wise *P* value ranges - all of the 11 QTL were significant at FDR *P* value <0.1 in at least one environment

^f^Stripe rust IT and severity from the field experiments and response to *Pst* races to which the marker showed significant association at FDR-adjusted probabilities

^g^References given in the text, RAF = Resistance allele frequency, Chr. = chromosome

PLM_IT_12 = Pullman, infection types, 2012

PLM_IT_14 = Pullman, infection types, 2014

PLM_DS_12 = Pullman, disease severity, 2012

PLM_DS_14 = Pullman, disease severity, 2014

MTV_IT_12 = Mount Vernon, infection types, 2012

MTV_IT_13 = Mount Vernon, infection types, 2013

MTV_IT_14 = Mount Vernon, infection types, 2014

MTV_DS_12 = Mount Vernon, disease severity, 2012

MTV_DS_13 = Mount Vernon, disease severity, 2013

MTV_DS_14 = Mount Vernon, disease severity, 2014

ETH_IT_12 = Ethiopia, infection types, 2012

ETH_IT_14 = Ethiopia, infection types, 2014

ETH_DS_14 = Ethiopia, disease severity, 2014

Among the 11 highly significant genomic regions, a block of haplotypes covering a genetic distance from 65 to 74 cM on chromosome 1B was identified to harbor major *Pst* resistance QTL/genes. SNPs *IWB11553* (mapped at 65 cM) and *IWB9661* (mapped at 74 cM) represented loci significantly associated with stripe rust IT and DS on 1BS (Table 2). The phenotypic variation explained by *IWB11553* and *IWB9661* loci were in the range of 3.1–9.6% and 3.4–6.7%, respectively.

On the short arm of chromosome 2A, a cluster of significant SNPs covering a genetic distance of 9.4–47.0 cM was detected. Among these, *IWB11136* (mapped at 9.4 cM), *IWB6584* (mapped at 26 cM) and *IWB57199* (mapped at 47.0 cM) showed strong associations (FDR *P* < 0.1) across multiple locations and growing seasons. The phenotypic variation explained by *IWB11136*, *IWB6584* and *IWB57199* loci ranged from 3.5–8.3%, 3.1–11.2% and 3.4–9.5%, respectively. Each SNP was in strong LD with each other (*r* ^*2*^ = 0.60, 0.68 and 0.88 between *IWB11136* and *IWB6584*, *IWB11136* and *IWB57199*; and *IWB6584* and *IWB57199*, respectively), in spite of extended genetic distance in the consensus map.

On chromosome 4A, SNP *IWB24187* (located 114.5 cM) was among the 11 strongly associated markers representing effective stripe rust resistance loci. The amount of variation explained by *IWB24187* ranged 2.8–6.1%. On chromosomes 5A, SNP markers *IWA1280* and *IWB28837*, mapped at 15.7 cM and 123 cM, were significant with *R* ^*2*^ values in the range of 2.7–6.1 and 3.0–7.1%, respectively. Several other SNP markers that were mapped close to *IW1280* and *IWB28837* were also significant at nominal probability of association (*P* < 0.005 across multiple locations and growing seasons), but unable to pass the stringent criteria based on FDR-adjusted *P* < 0.1.

On the long arm of chromosome 6A, SNP *IWB69846* (mapped at 136.9 cM) also showed strong association with *Pst* IT and DS. The amount of variation explained by the resistance loci associated with *IWB69846* ranged from 3.1–8.3%. Two resistance loci were identified on chromosome 7B, associated with SNP markers *IWA3506* (mapped on the short arm at 57 cM) and *IWB58601* (mapped on the long arm at 155.4 cM). The resistance locus tagged by *IWA3506* was detected as significant both in the seedling and adult plant tests with an average *R* ^*2*^ value of 6% at adult stage and 11.4% at seedling stage. The resistance locus tagged by *IWB58601* was effective across multiple locations and seasons at adult plant stage and showed average *R* ^*2*^ value of 4.8%.

Seedling resistance to *Pst* detected by GWAS among the Ethiopian wheat accessions is summarized in Table 2. Associations significant at FDR-adjusted *P* value <0.1 were detected for eight genomic regions on chromosomes 1B, 2A, 2B, 2D, 5B and 7B. The phenotypic variation explained by each of the eight regions ranged from 9.4 to 13.8%. Among these, *IWB9661* (mapped to chromosome 1B at 74.4 cM), *IWB6584* and *IWB57199* (mapped to chromosome 2A at 26 cM) and *IWA3506* (mapped to chromosome 7B at 56.9 cM) were commonly detected in the seedling and adult plant tests. On chromosome 2B, SNP *IWB43797* (located at 134.5 cM) was significantly associated with seedling resistance to PSTv-41 and PSTv-106. On chromosome 2D, SNP *IWB10441* showed strong association with resistance to PSTv-106. Two FDR *P* < 0.1 significant genomic regions were detected on chromosome 5B for seedling resistance to *Pst*, which were represented by SNPs *IWB14332* (mapped at 29.1 cM) and *IWB48375* (mapped at 72 cM). The LD between the two genomic regions was non-significant (*r* ^*2*^ < 0.001), indicating that they represent two different resistance loci.

### Mapping of seedling resistance to Pgt

GWAS identified nine genomic regions significantly associated with seedling response to the four tested races of *Pgt* (Table 3). Among these, a resistance locus mapped to the short arm of chromosome 2B was significantly associated with response to race TTKSK. This locus was represented by SNP *IWB26389*, which was mapped at 99.9 cM on 2BS. *IWB26389* explained 8.0% of the phenotypic variance in the germplasm panel. One additional genomic region on 7B, *IWB64169* at 171.1 cM, was significantly associated with race TTKSK. This genomic region accounted for about 2.4% of the total variation in IT response against TTKSK. GWAS of seedling resistance to race TTTTF identified a single genomic region (represented by *IWB34733*) that was mapped to the long arm of chromosome 4A at 144.4 cM. The phenotypic variance explained by *IWB34733* was 15%. The locus tagged by *IWB34733* was also effective against race TKTTF. Five genomic regions were significantly associated with seedling resistance to race TRTTF. These putative resistance loci were mapped to chromosomes 1B, 2A, 3B, 6A and 7B. The five genomic regions were tagged by *IWB39306* (chromosome 1B at 60.6 cM), *IWB12320* (chromosome 2A at 106.3 cM), *IWB19479* (chromosome 3B at 136.3 cM), *IWA5416* (chromosome 6A at 5.6 cM) and *IWB2378* (chromosome 7B at 92.5 cM). The phenotypic variance explained by the genomic regions significant for race TRTTF ranged 5.1% to 6.4%. On chromosome 6B, a genomic region tagged by SNP marker *IWB14375* showed highly significant association with response to race TKTTF. This genomic region was responsible for 9% of the phenotypic variation. *IWB14375* was significantly associated also with resistance to races TTTTF and TRTTF.

**Table 3:** Genomic regions significantly associated with seedling responses to the four races of *Puccinia graminis* f. sp. *tritici* in the Ethiopian bread wheat accessions

QTL-tagging SNP index	SNP	Associated SNPs index	Chr.	Position (cM)	MAF	*P* value	*R* ^*2*^ (%)	FDR *P* values
TRTTF								
*IWB39306*	**A**/G	–	1B	60.6	0.1	1.30E-04	5.08	*
*IWB12320*	**T**/C	–	2A	106.3	0.15	3.40E-05	5.98	*
*IWB19479*	**T**/G	*IWB1595*	3B	136.3	0.07	7.20E-05	5.47	*
*IWA5416*	**T**/C	*IWB11315, IWB22036, IWB60233, IWB67415*	6A	5.6	0.08	2.00E-05	6.35	*
*IWB2378*	T/**C**	*IWB4726, IWB4725, IWB7743, IWB65791*	7B	92.5	0.19	2.00E-05	6.35	*
TTKSK								
*IWB26389*	**A**/G	*IWB42742, IWB1518, IWB24614, IWB32327*	2B	99.9	0.31	2.40E-10	8.02	****
*IWB64169*	**A**/G	*IWB63221*	7B	171.1	0.13	3.00E-04	2.41	**
TTTTF								
*IWB34733*	A/**G**	*IWB63979, IWB61381, IWB59018*	4A	144.4	0.08	8.90E-10	14.62	****
TKTTF								
*IWB14375*	T/**G**	*IWB59006, IWB72471, IWB59005*	6B	113.7	0.096	3.60E-07	0.09	**

****= <0.0001

***< 0.001

**< 0.05

*< 0.1

### Validation of previously mapped rust resistance genes

Genotyping of the accessions with molecular markers linked to previously mapped stripe rust and stem rust resistance genes/QTL revealed the presence of *Lr34/Yr18/Sr57*, *Yr17/Sr38*, *Sr31/Yr9/Pm8/Lr26*, *Sr2/Yr30*, *Sr36* and *Sr24* at a frequency of 12.0%, 6.2%, 9.0%, 13%, 30% and 2.6%, respectively (Additional files 3 and 6 **)**. A single accession (Alidoro) was positive for the resistance-associated alleles of both *Lr34/Yr18/Sr57* and *Yr17/Sr38*, while four lines (Sofumer, Tusie, Bobicho and AMNA-4) carry both *Lr34/Yr18/Sr57* and *Sr31/Yr9/Lr26*. Three accessions (Sofumer, Bonny, and 231,658) carried both *Lr34/Yr18/Sr57* and *Yr30/Sr2*, while only one accession (KBG-01) was positive for the resistance-associated alleles of both *Yr17/Sr38* and *Yr30/Sr2*. Four accessions identified as landraces (231,658, 232,034 and 232,014) carried the resistance alleles of both *Yr30/Sr2* and *Sr36*. Accession 231,658 carried *Lr34/Yr18/Sr57*, *Yr30/Sr2* and *Sr36*. None of the accessions were positive for the *Lr67/Yr46* gene. Association analysis revealed a significant association of the *Lr34/Yr18* KASP marker wMAS000003 with field resistance to stripe rust at marker wise *P* value <0.005 in at least half of the test environments. However, no significant SNPs were detected on this region of chromosome 7D and none of the SNPs on the 7D chromosome were in significant LD with the KASP marker. Significant associations were also detected for *Yr17/Lr37/Sr38* KASP marker together with several other SNP markers including *IWB11136*, *IWB6584*, *IWB42693*, *IWB30196*, *IWB57199* and *IWB63394* that were in LD with the *Yr17* marker *Ventriup-LN2*.

Although there was no significant MTA detected for *Yr9* KASP marker 1RS.1BL_6110 in association with *Pst* resistance, the associated *Sr* gene (*Sr31*) showed an effect of reducing IT responses to races TRTTF, TTTTF and TKTTF by 44%, 50% and 45%, respectively. As expected, accessions postulated to carry the resistance allele of *Sr31* were highly susceptible to TTKSK. The resistance-associated allele of the marker for *Sr24* was carried by cultivars Huluka, Shorima, Hoggana, Millennium, and Kulkullu in this panel, and showed highly resistant responses to all races of the stem rust pathogen. Accessions carrying *Sr36* showed highly effective responses to TTKSK, but were mostly susceptible to TKTTK and TTTTF.

## Discussion

### Phenotypic variability and molecular diversity of the Ethiopian bread wheat accessions

A better understanding of the extent of genetic diversity and genetic basis of the responses to rust diseases in wheat may offer the prospect of effective exploitation of genetic resources and enhanced breeding for durable rust resistance in wheat. To this end, the current study surveyed Ethiopian bread wheat landraces and cultivars for resistance to *Pst* and *Pgt* under diverse environments and variable pathogen populations. Our data revealed that the accessions, most notably the landraces, possess considerable variation for resistance to both diseases. The sources of resistance identified with high levels of combined resistance to stripe rust and stem rust may be used as parental breeding lines for improving wheat resistance to these diseases. Profiling of the accessions with high-density SNP markers facilitated analyses of genome-specific molecular diversity and phylogeny between the landraces and elite cultivars as well as a genome-wide survey of marker-trait associations. It was evident that the landrace accessions were characterized by high levels of molecular diversity as measured by Nei’s gene diversity index, PIC values, NJ clustering algorithms and kinship analyses. By contrast, the Ethiopian bread wheat elite cultivars show high genetic similarity, with some of them nearly genetically redundant. Some of these nearly genetically redundant cultivars were released by different breeding programs and are widely grown across Ethiopia, which demonstrates one of the likely reasons for the frequent failures of deployed resistance genes and highlights the continued risk of disease epidemics due to minimal diversity. The loss of genetic diversity in modern wheat varieties compared to landraces has been reported by previous studies [19, 20, 53]. Such erosion of genetic variation in the elite wheat varieties may constrain the capacity to counter threats from changing pathogen populations. A particular concern in this regard is the fact that the highlands of Ethiopia are ‘hot spot’ areas for the *Triticum*-*Puccinia* pathosystem [16, 54, 55] and therefore the presence of high inoculum pressure and rapid changes in pathogen virulence. Cultivation of genetically uniform wheat cultivars and the practice of frequent double cropping in some wheat growing areas of the country may result in rapid selection for virulent races of the pathogen. The results of our analyses of genetic diversity in Ethiopian bread wheat cultivars highlight the continued risk of wheat rust epidemics due to the genetic vulnerability of the current wheat varieties and suggest the utilization of more genetic diversity to broaden the genetic base of elite wheat cultivars. We also believe that Ethiopian landraces are a unique source of unexploited diversity for wheat improvement [27].

### MTAs for Pst resistance and co-localization of the putative QTL with previously identified Yr genes/QTL

The mixed model analyses of association between the high-density genome-wide SNP markers and response to *Pst* in the Ethiopian bread wheat accessions highlighted several chromosome regions harboring putative resistance loci. Eleven FDR *P* < 0.1-significant regions, perhaps of large effect, were identified when employing the most stringent criteria. Four additional genomic regions were significantly associated (FDR-adjusted *P* < 0.10) with seedling resistance to stripe rust. Aiming to keep a reasonable power of statistical association and identify loci conferring intermediate effects, less stringent GWAS tests based on marker-wise significance tests were also considered. The latter approach was supported by associations detected across multiple location and seasons. Fifty-six additional *Pst* resistance loci, at a marker-wise *P* < 0.005 in at least half of the test environments, were detected. The amount of variation explained by the 67 genomic regions ranged from 54.3 to 52.1%. The reason for the partial account of the variation in the field response to stripe rust by the significant markers could be attributed to the fact that the current GWAS methods are more suited to the identification of common variants of large to intermediate effects [56]. In addition, the 90 K iSelect chip did not provide enough marker coverage in the D genome to identify all *Pst* resistance loci present in the germplasm panel. It was evident that none of the 90 K SNP markers mapped to the short arm of chromosome 7D were linked to the marker for known *Pst* resistance gene *Yr18* (*Lr34*/*Yr18*/*Pm38*) included as a control in a separate GWAS that showed experiment-wise significant associations with *Pst* resistance at multiple locations.

The percent of variation explained by the 56 MTAs from the field experiment support that many are unlikely false-positives**.** Nonetheless, the details of only the 11 highest-confidence resistance-associated MTAs from the field experiment and four MTAs from the greenhouse seedling resistance screening are further discussed.

To determine whether any known resistance genes coincided with the 15 putative resistance regions identified in this study, the current results were compared with the integrated genetic maps of *Pst* resistance genes and SNP markers constructed by Maccaferri et al. [24] and Bulli et al. [26]. The genomic regions harboring eight of the eleven loci identified from our field studies of stripe rust resistance overlap with positions of previously reported QTL. Among the three potentially novel resistance loci, *IWB28837* and *IWA1280* were associated with resistance on chromosomes 5A at 15.7 and 89.6 cM positions, respectively. In line with their distant map positions, *IWB28837* and *IWA1280* are not in LD with each other, and thus tag distinct genomic regions. The confidence intervals of MTAs tagged by these two SNPs do not overlap with positions of any of the previously reported QTL or *Yr* genes. Thus, *IWA1280* and *IWB28837* likely represent novel resistance loci for *Pst*. On the short arm of 7B, *IWA3506* (at 57.0 cM) was significant in both seedling and adult plant tests. *Yr63* has been mapped to the short arm of 7B chromosome (McIntosh et al., 2013). Based on the relative genetic position of *Yr63* in the integrated genetic map constructed by [24], *IWA3506* was mapped at an average genetic distance of 30.0 cM from *Yr63*, which indicates that *IWA3506* is likely different from *Yr63*, and thus represents a newly documented stripe rust resistance locus.

**Fig. 7:**
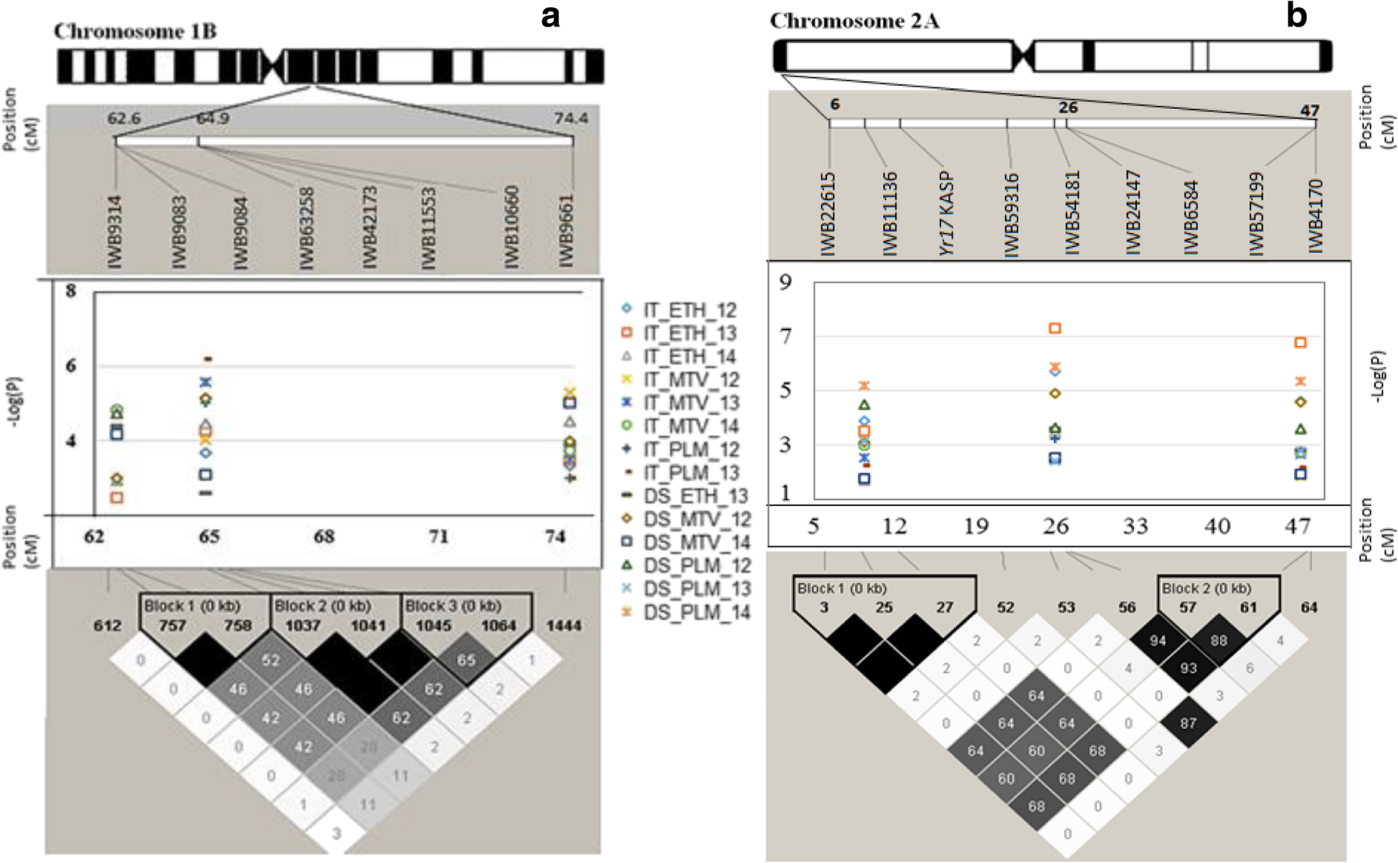
Linkage disequilibrium patterns of haplotype blocks of SNP markers significantly associated with stripe rust resistance. **a** On the long arm of chromosome 1B and (**b**) on the short arm chromosome 2A. The upper part of the graph show genetic distance (cM) from the 90 K SNP consensus map ([36]. The middle part of the graph show -log (*P*) values of marker-trait associations for infection types (IT) over eight environments and disease severity (DS) over six environments plotted against genetic distance (cM). The lower part of the graph shows local LD *r* ^*2*^ value patterns. Values within the diamonds of the triangular LD matrix are the *r* ^*2*^ values multiplied by 100. IT_ETH_14 = infection type (IT) Ethiopia 2014, IT_ETH_12 = IT Ethiopia 2012, IT_ETH_13 = IT Ethiopia 2013, IT_MTV_14 = IT Mount Vernon 2014, IT_MTV_13 = IT Mount Vernon 2013, IT_MTV_12 = IT Mount Vernon 2012, IT_PLM_14 = IT Pullman 2014, IT_PLM_12 = IT Pullman 2012, DS_ETH_13 = Disease severity (DS) Ethiopia 2013, DS_MTV_14 = DS Mount Vernon 2014, DS_MTV_12 = DS Mount Vernon 2012, DS_PLM_14 = DS Pullman 2014, DSPLM_12 = DS Pullman 2012, DS_PLM_13 = DS Pullman 2012

The haplotype blocks of significant stripe rust resistance loci detected on chromosome 1B spanning a segment of 63–74 cM likely harbor more than one resistance locus. Figure 7a illustrates LD relationships between the two loci on 1BS (*IWB11553* at 64.9 cM and *IWB9661* at 74.4 cM), which were all less than *r* ^2^ = 0.03. This indicates that *IWB11553* and *IWB9661* likely represent distinct genomic regions. Chromosome 1B is rich with *Yr* genes/QTL, and a number of *Yr* genes have been mapped to the proximity of the genomic regions tagged by *IWB9314*, *IWB11553* and *IWB9661*, including *Yr15*, *Yr24/Yr26/YrCh42*, *YrAlp*, *YrCH52*, *Yr52*, *Yr64* and *Yr65*. Other *Pst* resistance QTL were also previously mapped in this region of chromosome 1B, including *QYr.caas-1BL.1RS_SHA3/CBRD* and *QYr.cim-1BS_Pastor* [57–61]. Additional research is needed to fully characterize chromosome 1B resistance.

We detected significant associations between stripe rust resistance and SNP markers mapped close to the translocation segment harboring *Yr17* including the *Yr17* marker *Ventriup-LN2*. Since virulence to *Yr17* is widespread [29, 30], the significant association of *Yr17* and other SNP markers may indicate the presence of another effective resistance gene tightly linked to *Yr17*. Among the three significant SNPs detected, *IWB11136* (located at 9.4 cM) was in perfect LD (*r* ^*2*^ = 1.0) with the *Yr17* KASP marker. *IWB6584* and *IWB57199* were mapped at the 26 and 47 cM positions, respectively and in LD with the *Yr17* KASP marker with *r* ^*2*^ value of 0.64 and 0.68, respectively (Fig. 7b **)**. Hence, *IWB6584* and *IWB57199* may not be located on the 2NS.2AS translocation segment carrying *Yr17*, but tightly linked and likely represent a gene responsible for the high level of resistance exhibited by lines positive for the favorable alleles of the SNPs. Several other temporarily designated QTL have been identified on the short arm of chromosome 2A, including *QYr.sun-2A_Wollaroi* [62], *QYr.tam-2AS_TAM111* [63], *QYrva.vt-2AS_VA00W-38* [64], *QYr.inra_2A1_Recital* [65] and *QYr.ucw-2A_PI610750* [66]. *IWB6584* and *IWB57199* could be related to these QTL. Tan et al. (2013) reported mapping of a QTL on 2AS encoding 12-oxo-phytodienoic acid reductase (OPR) that is collinear to an *Yr17*-containing-fragment of the chromosome arm. The genes encoding OPR are known to play critical roles in insect and disease resistance pathways in higher plants [67, 68]. Further genetic analysis is required to determine the relationship of MTAs detected by *IWB6584* and *IWB57199* with previously mapped genes/QTL.

On chromosome 6A, an MTA associated with field resistance to *Pst* was identified at FDR-adjusted probability *(P*) <0.1 at multiple locations and seasons. This locus was tagged by the SNP *IWB69846* at the map position of 136.9 cM. Previously, *YrLM168* [69] and other temporarily designated QTL [*QYr-6A_Avocet* [70], *QYr-6A_Saar* [71], *QYr.ufs-6A_Kariega* [72] and *QYr.cim-6AL_Francolin* [73] have been mapped to the vicinity of *IWB69846*, and could be related to the locus tagged by *IWB69846*. On chromosome 4A, *IWB24187* represents an MTA mapped closely to *Yr51* [74] and two other QTL, *QYr-4A_Sachem* [75] and *QYrst.orr-4AL_Stephens* [76]. Further genetic analysis will be required to determine the relationship between the resistance locus tagged by *IWB24187* and the previously mapped QTL/genes.

On the long arm of chromosome 7B, SNP marker *IWB58601* was associated with *Pst* resistance across multiple field tests. The confidence interval of the genomic region tagged by *IWB58601* overlaps with the map positions of *Yr59*, *YrC591*, *Yr52*, *Yr67*, *YrZH84* and QTL, *QYr.cim-7BL_Pastor*, *QYr7BL_Strongfield* [58, 77, 78]. *Yr52* and *Yr59* confer high-temperature adult-plant (HTAP) resistance to stripe rust, while *YrC591*, *Yr67* and *YrZH84* are dominant genes conferring seedling resistance to stripe rust. It has been reported that *IWB37096* and *IWB71995*, both mapped at 148.7 cM on 7BL [36], are flanking *Yr67* with a respective genetic distance of 1.1 and 0.6 cM [79]. In the present study, these two SNPs (*IWB37096* and *IWB71995*) were not among the 67 significant MTAs and in non-significant LD with *IWB58601*, suggesting that the QTL tagged by *IWB58601* may be different from those of *IWB37096* and *IWB71995* as well as *Yr63*. However, since these SNPs and the previously mapped resistance genes are located close to each other, further characterization and genetic analysis is required to determine whether they are distinct from those previously mapped resistance genes.

### MTAs for Pgt resistance and co-localization of the resistance loci with previously identified *Sr* genes/QTL

Five genomic regions were significantly associated with seedling resistance to race TKTTF. Among these, *IWB39306* was mapped close to the segment of 1BS that harbors the 1BL.1RS rye translocation carrying *Sr31* [80]. *Sr31* is ineffective to TTKSK, but confers effective resistance to TRTTF and TKTTF [16, 33]. To determine if *IWB39306* is related to *Sr31*, we genotyped the accessions with a KASP marker for 1BL.1RS and performed LD analysis. *IWB39306* and the KASP marker for 1BL.1RS were in complete LD, indicating that *IWB39306* is detecting allelic variation based on the presence or absence of 1BL.1RS. The accessions carrying the resistance-associated allele of *IWB39306* are all elite Ethiopian cultivars. Working on the North American spring wheat breeding germplasm, Bajgain et al. [81] identified 13 significant SNPs on the short arm of chromosome 1B (position range 44–65 cM) that also showed various levels of significant associations with resistance to race TRTTF in our study, but did not pass the FDR threshold of <0.1. We found strong LD between the SNP markers identified by Bajgain et al. [81], *Sr31* and *IWB39306* (*r* ^*2*^ ranging from 0.41 to 1.00). This further clarifies the uncertainty whether the 13 significant markers are linked to *Sr31*, or to a novel gene of resistance to TRTTF.

The SNP locus *IWB12320* on chromosome 2A at 106.3 cM was significantly associated with response to race TRTTF. Eighteen of the 25 accessions carrying the resistance-associated allele of *IWB12320* are landraces. Stem rust resistance gene *Sr21* was mapped approximately 50 cM from the centromere on the long arm of 2A [82, 83]. *Sr21* confers resistance to several races of *Pgt*, including those in the Ug99 group. However, *Sr21* is derived from *T. monococcum* and was later transferred to hexaploid wheat [82]. Hence, *Sr21* is not common in landrace accessions, and suggests that *IWB12320* is tagging a resistance locus or allele distinct from *Sr21*. Stem rust resistance genes *Sr48* and *SrTm4* have also been previously mapped to the long arm of chromosome 2A [84, 85]. However, *IWB12320* may not be related to *SrTm4*, as the latter was identified from the diploid wheat, *Triticum monococcum*, and is not common in hexaploid wheat. *Sr48* (first designated as *SrAnl*) is a seedling resistance gene that was mapped to chromosome arm 2AL of the bread wheat cultivar Arina. Based on the genetic map position of *Sr48* (~55 cM from the centromere), it is likely that the genomic region tagged by *IWB12320* corresponds to *Sr48*. Allelism tests will be required to test the relationships between *IWB12320* and *Sr48*.

On 3BL, SNP *IWB19479* (mapped at 136.3 cM) identified a genomic region significantly associated with the response to race TRTTF. This locus may represent a new resistance gene since no race TRTTF-effective seedling resistance genes have been mapped to chromosome 3B so far. Similarly, no TTKSK-effective seedling resistance genes have been identified on chromosome 7B [86]. Therefore, the significant genomic region detected on chromosome 7B in the present study (*IWB2378* mapped at 92.5 cM) is likely tagging a new resistance gene effective to TTKSK. On the short arm of chromosome 6A, SNP *IWA5416* (mapped at 5.6 cM) tagged a genomic region significantly associated with resistance to TRTTF. This 6AS locus is most likely linked to the gene *Sr8a*, which is effective to race TRTTF [87, 31]. Bajgain et al. [81] identified a significant association of *IWA5416* and 56 other SNP markers with race TRTTF, validating the association of this genomic region with resistance to *Pgt*.

Analysis of response to TTTTF identified a significant genomic region that was mapped to long arm of chromosome 4A. This locus was identified by SNP *IWB34733*, mapped at 144.4 cM, and was also effective to TTKSK and TRTTF, but did not pass the FDR threshold of significant associations for these races. It is likely that *IWB34733* is related to *Sr7a*. Bajgain et al. [81] also identified 51 significant SNPs mapped on 4AL, spanning 142–164 cM, in North American spring wheat breeding germplasm, one of which was *IWB34733*.

A single genomic region was detected that was significantly associated with race TKTTF, a newly detected Ethiopian *Pgt* race that defeated resistance in the popular cultivar ‘Digalu’ [16]. SNP marker *IWB14375*, mapped at 113.7 cM on 6B, identified this genomic region. This region of chromosome 6B is known to harbor *Sr11*, which is effective against TKTTF [86]. Bajgain et al. [81] also detected significant association of *IWB14375* with response to race TKTTF in North American spring wheat germplasm. Nirmala et al. [88] identified seven SNP markers linked to *Sr11*. Among the seven SNP markers, *IWB59006* and *IWB72471* also showed highly significant association with the response to TKTTF in the present study. Both *IWB59006* and *IWB72471* were in strong LD (*r* ^*2*^ = 0.8–1.0) with *IWB14375*, indicating that the locus tagged by *IWB14375* corresponds to *Sr11*.

A genomic region on the short arm of chromosome 2B, tagged by *IWB26389*, was significantly associated with IT response to race TTKSK. Although the short arms of 2A, 2B and 2D are known to carry *Sr32* [79], it is unlikely that the three markers represent *Sr32* as it is not common in landrace accessions due to its origin from *Ae. speltoides* and relatively recent translocations to hexaploid wheat. The stem rust resistance gene *Sr36* derived from *Triticum timopheevi* is also located on 2BS and confers resistance against TTKSK [89]. To determine if *IWB26389* is associated with *Sr36*, we determined LD between the KASP marker for *Sr36* (wMAS000015) and *IWB26389*. The result showed that *IWB26389* and *Sr36* KASP marker are in complete LD, which indicates that *IWB26389* is also tagging the *T. timopheevi* segment that harbors *Sr36.*

We identified a genomic region on 7BL, tagged by *IWB64169* at 171 cM, significantly associated with response to race TTKSK. Although there are several QTL effective to Ug99 on chromosome 7B, no effective seedling resistance gene has been identified so far [86]. Therefore, the significant genomic region detected on 7BL in the present study may be a new resistance gene effective to TTKSK. Based on LD analysis, *IWB64169* represents a different genomic region from *IWB2378*, which was significant to race TRTTF.

## Conclusion

The limited pool of alleles present in the Ethiopian bread wheat cultivars may offer narrow perspective for germplasm improvement and consequently limit the capacity to deal with threats from wheat rust epidemics. Notwithstanding, enough genetic variation resides within the landrace accessions that can be utilized to broaden the genetic base of rust resistance in wheat breeding germplasm. In addition to characterizing the genetic diversity of the Ethiopian bread wheat cultivars and landraces, this study highlights the potential of GWAS to accelerate the translation of landrace germplasm diversity toward applied wheat improvement. Several newly documented resistance loci were discovered from Ethiopian landraces, along with detection of previously reported resistance genes. The molecular markers linked to resistance loci identified in the current study can be used to efficiently select for resistance to diversify the genetic basis of elite cultivars.

## Additional files

Additional file 1: Virulence/avirulence formula of *Puccinia striiformis* f. sp. *tritici* isolates used for seedling resistance screening of the Ethiopian wheat accessions. (XLSX 10 kb)

Additional file 2: Pearson’s correlation coefficients among stripe rust infection type (IT) and disease severity (DS) in each location and season. (XLSX 12 kb)

Additional file 3: Infection types and disease severity observed on Ethiopian wheat cultivars and landraces tested with races of *Puccinia striiformis* f. sp. *tritici* and *Puccinia graminis* f. sp. *tritici* at seedling stage and evaluated under field conditions. Allele report for markers linked to previously known stripe rust and stem rust resistance genes and SNP markers significantly associated with the resistance to both diseases (identified in this study) are summarized. (XLSX 50 kb)

Additional file 4: Comparison of different association test models using Quantile-Quantile (Q-Q) plots using BLUP values of stripe rut IT and DS across all environments. The Q-Q plot determines the magnitude of the deviation of the observed association between the markers and response to stripe rust from the expected null hypothesis of no association. Under the assumption of no association between the SNPs and the traits, a large inflation of observed *P* values from the expected *P* values indicates spurious associations. Only few true SNP-trait associations are expected to deviate from the null hypothesis. (DOCX 95 kb)

Additional file 5: Genomic regions significantly associated with field resistance of the Ethiopian bread wheat accessions to *Puccinia striiformis* f. sp. *tritici* at marker wise *P* < 0.005 in at least four of the eight environments. (XLSX 15 kb)

Additional file 6: Frequency of favorable alleles of the molecular markers linked to previously mapped stripe rust and stem rust resistance genes/QTL in the Ethiopian wheat accessions. (DOCX 16 kb)

## Abbreviations

APR: Adult plant resistance
CMLM: Compressed mixed linear model
DS: Disease severity
GLM: General linear model
GWAS: Genome-wide association study
IT: Infection types
LD: Linkage disequilibrium
MAF: Minor allele frequency
MLM: Mixed linear model
MTA: Marker-trait association
PC: Principal component
PCA: Principal component analysis
Pgt: Puccinia graminis f. sp. tritici
PNW: Pacific Northwest
Pst: Puccinia striiformis f. sp. *tritici*
Q-Q: quantile-quantile
QTL: Quantitative trait locus
SNP: Single nucleotide polymorphism
