# Supplementary material for: Characterization of molecular diversity and genome-wide mapping of loci associated with resistance to stripe rust and stem rust in Ethiopian bread wheat accessions
Source: BMC Plant Biol. 2017 Aug 4;17:134. doi: 10.1186/s12870-017-1082-7 (PMC5545024; doi:10.1186/s12870-017-1082-7)
Supplement: Supplementary file 4 — Comparison of different association test models using Quantile-Quantile (Q-Q) plots using BLUP values of stripe rut IT and DS across all environments. The Q-Q plot determines the magnitude of the deviation of the observed association between the markers and response to stripe rust from the expected null hypothesis of no association. Under the assumption of no association between the SNPs and the traits, a large inflation of observed P values from the expected P values indicates spurious associations. Only few true SNP-trait associations are expected to deviate from the null hypothesis. (DOCX 95 kb) [file 12870_2017_1082_MOESM4_ESM.docx]

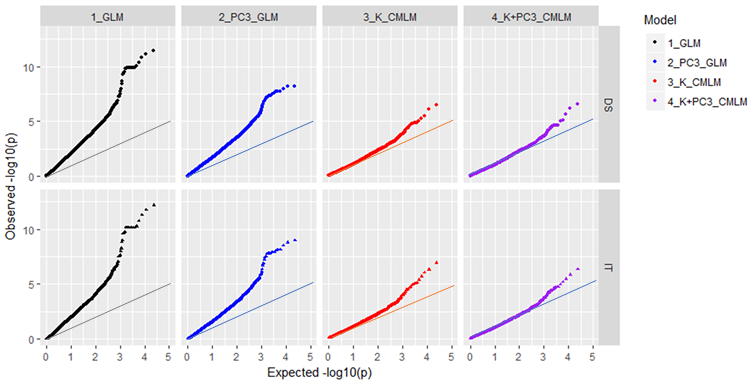


**Supplemental File S6.** **Comparison of different association test models using** Quantile-Quantile (Q-Q) plots using BLUP values of stripe rut IT and DS across all environments. The Q-Q plot determines the magnitude of the deviation of the observed association between the markers and response to stripe rust from the expected null hypothesis of no association. Under the assumption of no association between the SNPs and the traits, a large inflation of observed *P* values from the expected *P* values indicates spurious associations. Only few true SNP-trait associations are expected to deviate from the null hypothesis.
