# Supplementary material for: Characterization of molecular diversity and genome-wide mapping of loci associated with resistance to stripe rust and stem rust in Ethiopian bread wheat accessions
Source: BMC Plant Biol. 2017 Aug 4;17:134. doi: 10.1186/s12870-017-1082-7 (PMC5545024; doi:10.1186/s12870-017-1082-7)
Supplement: Supplementary file 6 — Frequency of favorable alleles of the molecular markers linked to previously mapped stripe rust and stem rust resistance genes/QTL in the Ethiopian wheat accessions. (DOCX 16 kb) [file 12870_2017_1082_MOESM6_ESM.docx]

**Supplemental File S5.** Frequency of favorable alleles of the molecular markers linked to previously mapped stripe rust and stem rust resistance genes/QTL in the Ethiopian wheat accessions.
